# Yield and Composition of the Essential Oil of *Clinopodium nepeta* subsp. *spruneri* as Affected by Harvest Season and Cultivation Method, i.e., Outdoor, Greenhouse and In Vitro Culture

**DOI:** 10.3390/plants12244098

**Published:** 2023-12-07

**Authors:** Georgia Vlachou, Maria Papafotiou, Dimitra J. Daferera, Petros A. Tarantilis

**Affiliations:** 1 Laboratory of Floriculture and Landscape Architecture, Department of Crop Science, School of Plant Sciences, Agricultural University of Athens, Iera Odos 75, 118 55 Athens, Greece; gvlachou@aua.gr; 2 Laboratory of Chemistry, Department of Food Science and Human Nutrition, School of Food and Nutritional Sciences, Agricultural University of Athens, Iera Odos 75, 118 55 Athens, Greece; daferera@aua.gr (D.J.D.); ptara@aua.gr (P.A.T.)

**Keywords:** *Calamintha nepeta* subsp. *glandulosa*, chemical profile, environmental variation, Lamiaceae, medicinal aromatic plant, Mediterranean native plant, phytochemical analysis, quality analysis, seasonal variation

## Abstract

*Clinopodium nepeta* subsp. *spruneri* is an aromatic herb with a mint-oregano flavor, used in Mediterranean regions in traditional medicine. The aerial parts of the plant are rich in essential oil that has antioxidant, antimicrobial and anti-inflammatory properties as well as insecticidal activity. The aim of our work was to determine the yield and composition of the essential oil of the plant, in relation to the harvest season and cultivation method, i.e., outdoor, greenhouse and in vitro culture, using gas chromatography-mass spectrometry (GC-MS) as an analytical tool. Essential oil yield fluctuated similarly in outdoor and greenhouse plants during the year (0.9–2.6%), with higher percentages (2.1–2.6%) in the hottest periods June–October (flowering stage) and April (vegetative stage), and was similar to the yield in in vitro plants (1.7%). More compounds were identified in the oil of outdoor and greenhouse plants (35) compared to that of in vitro plants (21), while the main compounds were the same, i.e., pulegone (13.0–32.0%, highest in February–April, 15.0% in vitro), piperitenone oxide (3.8–31.8%, lowest in February, 34.2% in vitro), piperitone epoxide (4.6–16.4%, highest in February, 15.5% in vitro), D-limonene (2.1–8.8%, lowest in February, 10.0% in vitro), isomenthone (2.3–23.0%, highest in February, 4.6% in vitro), germacrene D (1.9–6.5% highest in December-April, 2.9% in vitro) and dicyclogermacrene (2.1–5.3%, highest in December–April, 5.2% in vitro). Therefore, greenhouse and in vitro cultures were equally efficient in yielding essential oil and its constituents as outdoor cultivation, while in outdoor and greenhouse cultivations, the harvest season, mainly due to the prevailing ambient temperatures, affected the essential oil yield and its percentage composition.

## 1. Introduction

From ancient times to the present, medicinal plants have played a key role in traditional medicine and form the basis of health care in many cultures. Although there has been a decline in the use of herbal medicines due to their replacement by synthetic medicines, in recent years there has been an increasing shift of the world towards natural products [1,2,3,4,5,6].

The genus *Clinopodium* (syn. *Calamintha*) belongs to the Lamiaceae family and includes aromatic medicinal plants found scattered in Western, Southern and South-Eastern Europe, the Mediterranean region, North Africa, Central Asia [7,8,9,10] and North and Latin America [11]. Since ancient times, *Clinopodium* spp. have been used in the folk medicine of various countries against insomnia, depression, spasms and cramp pains [1,11,12,13] and for the disinfection and healing wounds [14] and insect bites [15]. Also, due to their pleasant smell between oregano and mint, they are used as a spice under the name “mentuccia” or “nepitella” in Italy and Northern Portugal [16]. The essential oil of many species of the genus is used to improve the taste and aroma of many pharmaceutical products and the antimicrobial activities of plant oils and extracts have a number of applications, including raw and processed food preservation, pharmaceuticals, alternative medicine and natural therapies [13,17,18,19,20,21,22,23]. Furthermore, the essential oil of the *Clinopodium* species presents antitumor, anticancer and antioxidant activities [24,25].

The species *C. nepeta* has two subsp., *nepeta* and *spruneri* (syn. *glandulosa*). *C. nepeta* subsp. *spruneri* (Boiss.) Bartolucci and F. Conti, syn. *Calamintha nepeta* subsp. *glandulosa* (Req.) P.W. Ball, grows in rocky areas of Western and Southern Europe from 0 to 1500 m altitude [26,27] and in North East England [7]. In Greece, *C. nepeta* subsp. *spruneri* is native to both southern and northern regions [28], in damp, shady places, stream banks, forests and woodlands, roadsides and abandoned fields; it is called “agriomenta” (meaning wild mint in Greek) or “fliskouni” and is used for the preparation of medicinal teas for the stomach and throat pain and kidney disorders [29]. The plant is known in many countries for its medicinal uses as a tonic, antiseptic, antispasmodic and diuretic [12,30]. It is also used as an ornamental plant.

The aerial parts of *C. nepeta* are rich in essential oils that have antioxidant [19,20], antimicrobial [14,31,32,33,34] and anti-inflammatory properties [12,19,32], as well as insecticidal activities [34]. *C. nepeta* is traditionally found as an aromatic substance [26] and can be applied as a fragrance for insect repellent products [35].

Several studies of *C. nepeta* essential oils have been reported in the literature, which clearly indicate the presence of a remarkable chemical diversity and great intraspecific variability [33,36,37,38,39]. There is a consensus that pulegone is the dominant constituent, associated with different components such as menthone and/or isomenthone [33,36,38,40]. The major components in the oils of the diverse chemotypes generally belong to the C-3 oxygenated *p*-menthans such as pulegone, menthone, isomenthone, piperitone and piperitenone with their oxides. Three chemotypes of oils can be distinguished in *Clinopodium* species and subspecies (with some exceptions): (I) the first and most abundant chemotype consists of pulegone as the main component associated with either menthone and/or isomenthone, menthol and its isomers (first variant) or with piperitenone or piperitone and piperitenone oxides (second variant), (II) the second chemotype is characterized by the predominance of piperitone oxide and/or piperitenone oxide and (III) the rarest chemotype is distinguished by the presence of carvone and 1,8-cineole [33]. In wild populations of *C. nepeta* subsp. *spruneri* from Greece, intra-population variation in essential oil composition has been demonstrated [36,37,39], with researchers reporting the coexistence of two chemotypes [41,42]. The main components of the first chemotype were pulegone, menthone, piperitenone and piperitone, while piperitone *cis*- and *trans*-oxides, limonene and piperitenone epoxide were noted for the second chemotype.

Pulegone is “Generally Recognized As Safe” (GRAS) by the international Food and Drug Administration since 1965, while in 1974, it was included by the Council of Europe in the list of artificial flavoring substances that can be temporarily added to food without risk to public health [43]. Therefore, it is widely used commercially as a flavoring agent, as a base ingredient for perfumes and for various pharmaceutical products, but also for the preparation of toothpaste and mouthwash [44].

The essential oil composition is related to many variables [45,46,47], such as the genotype [48] and the geographical area of production, with main parameters the altitude [49], the harvest year [50] as well as the extraction system used [51]. In medicine, aromatic herbs have high economic importance for their essential oil worldwide, such as *Salvia*, *Thymus* and *Lavandula* species, and it has been shown by many works that factors such as soil texture, organic matter and minerals, annual rainfall, temperature, altitude, affect both the essential oil yield and its composition [52,53,54]. Differences in environmental conditions induced differences in the essential oil composition of *C pulegium* that were more quantitative than qualitative [55]. The content of the main components of the essential oil of *C. nepeta* subsp. *spruneri* was found to vary with the growth stage of the plant (vegetative/flowering) and the plant organs (inflorescences, leaves, stems) [41]. Cultivated plants of *C. pulegium* in the vegetative and flowering stages were a significant source of pulegone, and in the fruiting stage a significant source of menthone [55].

In vitro cultures can be a valid alternative for the rapid production of large quantities of plant material with a similar chemical profile to the corresponding field cultured plants, regardless of climate factors and eliminating geographical boundaries [56], while at the same time, the natural ecosystem is protected from the illegal harvesting of native plants from the natural environment. The effect of in vitro culture and growth regulators on essential oil composition and yield has been studied in a number of aromatic and medicinal plants, including *Clinopodium* spp., and various effects on secondary metabolite production profiles compared with field grown donor plant have been reported [24,57,58,59,60]. Moreover, it has been shown that many bioactive compounds, including essential oil components, can accumulate in in vitro culture at higher concentrations than in plants cultivated in the field [59,61,62]. Quantitative and qualitative modifications to the production of plant secondary metabolites can be induced by modifying the culture medium and conditions [57,63,64,65]. Thus, the use of plant tissue culture for up-regulating metabolism pathways may create a source of homogeneous and well-defined product [66].

The aim of our work was to determine the yield and composition of the essential oil of *C. nepeta* subsp. *spruneri*, in relation to the harvest season during one year and the cultivation method, i.e., outdoor, greenhouse and in vitro culture, using gas chromatography-mass spectrometry (GC-MS) as an analytical tool. There is only one report on the chemical composition of *Calamintha nepeta* L. (subsp. not reported) essential oil from microplants grown in vitro and their comparison with the parent plants grown in the field, which showed quantitative differences between them [59]. There is no report, to our knowledge, on the yield and composition of *C. nepeta* essential oil from greenhouse plants. The study of seasonal variation of the yield and chemical composition of plant essential oils provides useful information on the appropriate time to harvest the plant material depending on the desired secondary metabolites. Further, the recording of essential oil yield and chemical composition in relation to the cultivation method provides useful information for alternative forms of production, minimizing geographical effects on the product and providing the ability to qualitatively and quantitatively modify the essential oil yield and its chemical composition.

## 2. Results and Discussion

### 2.1. Average Percentage Content and Seasonal Variation of Essential Oil Extracted from Outdoor, Greenhouse and In Vitro Plants

**Figure 1:**
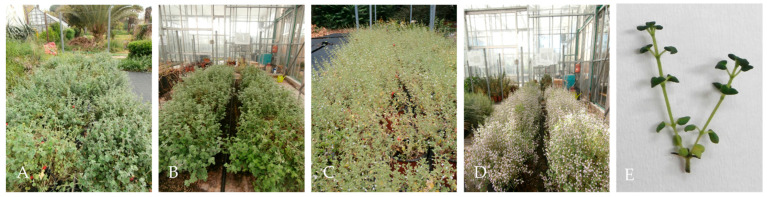
*C. nepeta* subsp. *spruneri* plants in the vegetative stage in outdoor (**A**) and greenhouse (**B**) cultivation just before the April harvest, at flowering stage in outdoor (**C**) and greenhouse (**D**) cultivation just before the June and August harvest, respectively, and in vitro cultivation at the stage of microshoot harvest (**E**).

**Figure 2:**
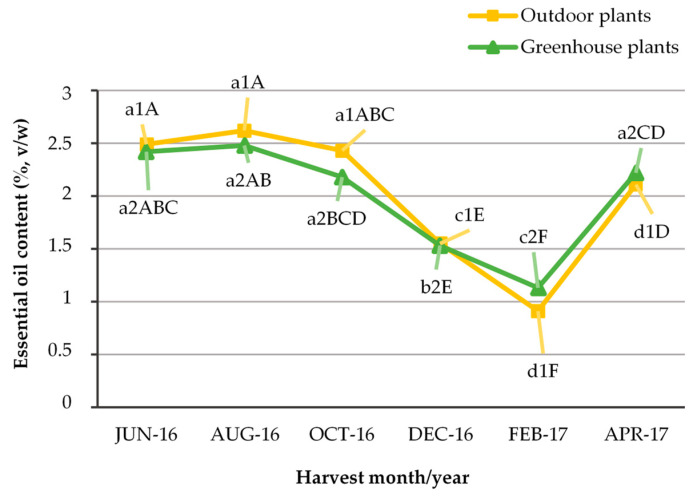
Percentage content of essential oil (ml essential oil/100 g dry sample) in outdoor and greenhouse plants of *C. netepa* subsp. *spruneri* at the different harvest seasons (months). ***: significant at *p* ≤ 0.001, NS: not significant at *p* ≤ 0.05; two-way ANOVA: *F*_interaction_: NS, *F*_harvest months_: ***, *F*_cultivation method_: NS; one-way ANOVA: a1, b1, c1…: comparison of means for outdoor plants (*F*: ***), a2, b2, c2…: comparison of means for greenhouse plants (*F*: ***), A, B, C…: comparison of means in both outdoor and greenhouse plants (*F*: ***).

**Figure 3:**
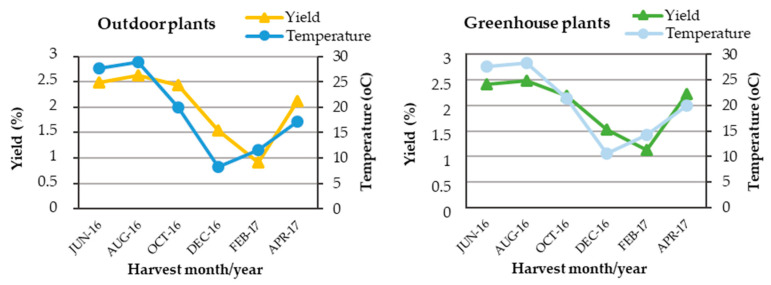
Percentage content of essential oil (ml essential oil/100 g dry sample) in outdoor and greenhouse plants of *C. netepa* subsp. *spruneri* at the different harvest seasons (months) vs average monthly ambient temperature outdoor and in the greenhouse.

In the June, August, October and December harvests, both outdoor and greenhouse plants were in the flowering stage, while in the February and April harvests, the plants were in the vegetative stage (Figure 1). The essential oil extracted from all kinds of plant material, outdoor, greenhouse and in vitro, was yellow in color. Its average percentage content ranged from 0.9 to 2.6% in outdoor plants depending on the harvest period and a similar variation was observed in greenhouse plants (1.2–2.5%) (Figure 2), while in in vitro microshoots it was 1.7%. The place of cultivation, i.e., outdoors or in the greenhouse, did not seem to significantly affect the essential oil yield, and this can be attributed to the fact that the greenhouse where cultivation took place was unheated with a cooling system for the summer months and therefore the temperatures were similar inside and outside the greenhouse (Figure 3), and it was glass covered, that allows for the passage of a high amount of radiation. There was an indication that outdoor plants had lower (0.9%) percentage content of essential oil in winter compared to the greenhouse plants (1.2%), and this reinforces the above comment about the temperature effect, as in winter the outdoor temperature was slightly lower compared to that of the greenhouse.

The essential oil percentage content was not affected by the cultivation method (cultivation site), but it was affected by the season of harvest in both outdoor and greenhouse plants (two-way ANOVA, Figure 2). The highest yield in outdoor and greenhouse plants was observed in the June, August and October harvest (2.3–2.6%), periods when plants were in the flowering stage and average ambient temperatures were above 20 °C (Figure 2 and Figure 3). In addition, a high yield of essential oil was also observed in the April harvest (Figure 2), a period when the plants were in the vegetative stage, so this may be due to the high average temperature of the month (above 20 °C), which was similar to the average October temperature (Figure 3). The lowest yield for both outdoor and greenhouse plants was observed in the February harvest (0.9% for outdoor and 1.2% for greenhouse plants) (Figure 2). This can be explained by the fact that these shoots were grown in the period January–February, when temperatures were the lowest of the year, while the shoots harvested in December (1.5% yield), although this month was colder than February, had grown in the November–December period, where temperatures were higher than the January–February period (Figure 3), and even allowed the plants to flower. The accumulation of plant secondary metabolites is highly dependent on a variety of environmental factors such as temperature, light, carbon dioxide, ozone, soil water, fertility and salinity [45]. In our experiments, outdoor and greenhouse plants were grown in pots with peat-perlite substrate (2:1 *v*/*v*), so ambient temperature and radiation seem to be the main reasons for variation in the essential oil yield in different harvest seasons. The effect of irradiance could be supported by the comparison of oil yield from in vivo (outdoor and greenhouse) and in vitro plants, as the yield of in vitro plants, which were grown at 25 ± 2 °C but under low irradiance (37.5 μmol m^−2^ s^−1^ provided by white fluorescent lamps) was in the range of the December yield in outdoor and greenhouse plants. Light is essential for the biosynthetic pathway of a growing plant. Key factors related to light radiation include intensity, photoperiod, direction and quality. Future in vitro experimentation providing different light quality, intensity and photoperiod could add scientific information on this issue, with the assumption that modification of the lighting conditions could support in vitro growth and proliferation of the species [67,68].

Similar yields to those given by the plants in our experiments have been reported in samples from wild populations of *C. nepeta* subsp. *spruneri*, during the flowering stage, collected from the Greek island Lefkada (2.0%) [36], Zonguldak in Turkey (2.7%) [69], Corinth in Northeast Peloponnese in Greece (3.3%) [39] and five regions of Central Italy (1.6–2.6%) [70]. However, there are several studies reporting a lower yield (0.1–1.2%) in flowering wild C. nepeta plants [34,37,71,72,73,74,75,76,77] in samples from France, Greece, Turkey, Italy, Croatia, Montenegro, Albania, Portugal and Tunisia. In addition, some researchers reported a wider range of essential oil yield from wild *C. nepeta* plants, in full flowering, e.g., Karousou et al. [42] from populations of both subspecies from Northern Greece and Crete (0.8–2.7%) and Negro et al. [78] in nine different populations of the nepeta subspecies from Southern Italy (1.2–3.0%).

Essential oil yield has been correlated with seasons, but mainly at the stage of plant growth determined by them, in various species of both the genus *Clinopodium* [41,55] and the family Lamiaceae, such as, e.g., *Mentha spicata* [79], *Ocium basilicum* [80], *Plectranthus amboinicus* [81], *Rosmarinus officinalis* [82,83], *Hypericum androsaemum* [84], *Salvia officinalis* [85], but also other medicinal plants, e.g., *Artemisia verlotiorum* (Asteraceae) [86] and *Santolina rosmarinifolia* (Asteraceae) [87]. In addition, there are several studies on the *Clinopodium* genus that report that the essential oil content also depends on exogenous factors such as the geographical area of harvest and the various soil and climate conditions that prevail [34,42,70,78,88]. The effect of climate conditions (temperature, relative humidity, rainfall, sunshine) has been reported for other medicinal species as well, such as *Lippia junelliana* [89], *Ocimum basilicum* [80], *Santolina rosmarinifolia* [87], *Achillea millefolium* [90] and *Tetradenia riparia* [91].

Regarding essential oil yield from in vitro cultures compared to wild growing plants or field cultures, information is conflicting. Comparing yields of non-flowering plants, in Mentha spicata field-grown and in vitro plants gave similar yields [92], in *Salvia fruticosa* in vitro plants gave higher yields [93], while in *Ocimum basilicum* field plants gave higher yields [94]. In field-grown flowering plants and non-flowering in vitro plants of *Eryngium planum* and *Lavandula viridis*, the essential oil yield of the field plants was higher and this was attributed to the flowering stage [95,96]. Finally, in *Ocimum basilicum* flowering plants, a greater yield was observed in field plants than in vitro [97]. In our experiments, the in vitro plants, being at the vegetative stage, gave a yield (1.7%) similar to the December one of the outdoor and greenhouse plants, which were at the flowering stage (Figure 2). While comparing the yield of in vitro plants with those of the February and April harvests, months when the outdoor and greenhouse plants were in the vegetative stage, the yield of the in vitro plants was higher than the yield of outdoor and greenhouse plants in February and lower than that of April (Figure 2). Thus, it appears that environmental factors such as temperature and irradiance affect essential oil yield more than the plant growth stage. The substrate could be another factor that affected the in vitro plant essential oil yield. We chose to use plain MS medium, i.e., without plant growth regulators, which ensured a stable response of the explants to the subcultures [67] that had to be used to ensure the required biomass for our analyses. The use of plant growth regulators, such as benziladenine, which has been shown in our previous work to promote proliferation in the species [68], was avoided, as it could show a carry-over effect from subculture to subculture, which could affect the quality of the microshoots, and become an additional factor of variability in essential oil yield and its chemical composition [98,99].

### 2.2. Chemical Composition, Seasonal Variation and Comparative Study of the Main Chemical Groups of Essential Oil Extracted from Outdoor, Greenhouse and In Vitro Plants

**Figure 4:**
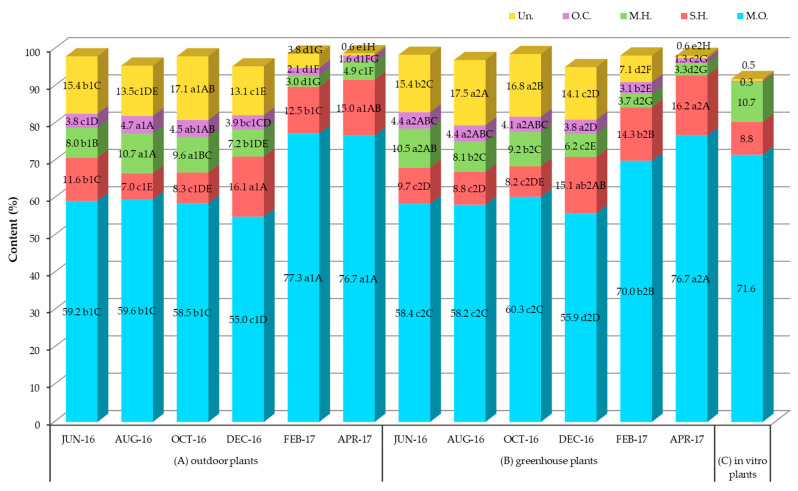
Dominant chemical groups of essential oils of (**A**) outdoor and (**B**) greenhouse *C. netepa* subsp. *spruneri* plants as affected by harvest season, as well as (**C**) in vitro plants. M.O. = monoterpene oxygenated, M.H. = monoterpene hydrocarbons, S.O. = sesquiterpen oxygenated, S.H. = sesquiterpen hydrocarbons, O.C. = other compounds, Un. = unknowns. Mean separation by Student’s *t*-test at *p* ≤ 0.05, *: significant at *p* ≤ 0.05, ***: significant at *p* ≤ 0.001. Monoterpene oxygenated: *F*_interaction_***, *F*_one-way ANOVA_***, monoterpene hydrocarbons: *F*_interaction_***, *F*_one-way ANOVA_***, sesquiterpen oxygenated: *F*_interaction_*, *F*_one-way ANOVA_***, Sesquiterpen hydrocarbons: *F*_interaction_*, *F*_one-way ANOVA_***, other compounds: *F*_interaction_*, *F*_one-way ANOVA_***, unknowns: *F*_interaction_***, *F*_one-way ANOVA_***; a1, b1, c1…: comparison of means for outdoor plants, a2, b2, c2…: comparison of means for greenhouse plants, A, B, C…: comparison of means of both outdoor and greenhouse plants.

The essential oil of *C. nepeta* subsp. *spruneri* in all three cultivation methods, outdoors, greenhouse and in vitro, was characterized by the presence of three dominant chemical groups, i.e., oxygenated monoterpenes (with main representatives: pulegone, piperitenone epoxide, isomenthone, piperitone epoxide), followed by monoterpene hydrocarbons (with main representative: *D*-limonene) and sesquiterpene hydrocarbons (with main representatives: germacrene D and dicyclogermacrene) (Figure 4). Similarly, in samples from other regions of Greece, i.e., the Island of Lefkada [36], Corinth in Peloponnese [39], Island of Zakynthos [41], Crete [42], but also in samples from Portugal [77], Croatia [100], Tunisia [34], Albania [76] and Turkey [101], the oxygenated monoterpenes were the main chemical group of the essential oil. On the contrary, in the essential oil of *C. nepeta* (subspecies was not reported) plants from Tuscany Islands in Central Italy, monoterpene hydrocarbons were reported as the main chemical group [59], verifying that various chemotypes of oils can be distinguished in *Clinopodium* species and subspecies [33].

Oxygenated monoterpenes were recorded in the different harvest seasons at a rate of 55.0–77.3% for outdoor plants, 55.9–76.7% for greenhouse plants and 71.6% for in vitro plants, followed by monoterpene hydrocarbons, 3.0–10.7% for outdoor plants, 3.3–10.5% for greenhouse plants and 10.7% from in vitro plants and sesquiterpene hydrocarbons, 7.0–16.1% for outdoor grown plants, 8.2–16.2% for greenhouse grown plants and 8.8% for in vitro plants (Figure 4).

Regarding the comparison of the concentrations of the main groups of the essential oil of outdoor and greenhouse plants, the two-way analysis showed a significant interaction between the main experimental factors, i.e., cultivation method and harvest season, and therefore we proceeded to compare the means of the experimental treatments. In plants grown outdoors, higher values of oxygenated monoterpenes, that represent the major chemical group, were recorded in February and April (77.3% and 76.7%, respectively) and the lowest value in December (55.0%) (Figure 4). Monoterpene hydrocarbons showed a higher value in August (10.7%) and lower values in February and April (3.0% and 4.9%, respectively), while sesquiterpene hydrocarbons showed higher values in December and April (16.1% and 15.0%, respectively) and lower values in August and October (7.0% and 8.3%, respectively) (Figure 4). Similar results were recorded for greenhouse plants, with the only difference being that monoterpene hydrocarbons showed a higher value, except in August and June (9.2% and 10.5%, respectively) (Figure 4). Cultivation site, i.e., outdoor or greenhouse, did not appear to significantly affect the percentage of the main chemical groups, and this may be attributed to the fact that the outdoor and greenhouse conditions were similar, as discussed above. Differences in the percentage of the main chemical groups were observed in our samples from both outdoor and greenhouse plants depending on the harvest season (Figure 3), as has been shown for other species of the Lamiaceae family [82,84,85] and other medicinal plants [86,87,90,91]. Oxygenated monoterpenes and sesquiterpene hydrocarbons had higher values during winter or early spring, when monoterpene hydrocarbons had the lowest values, the latter showing higher values in the June–October period and in vitro. The in vitro plants also had high values of oxygenated monoterpenes similar to the greenhouse ones. The seasonal variation of oxygenated monoterpenes and sesquiterpene hydrocarbons in our experiments confirms previous results in *Ocimum basilicum* in Egypt [80], where samples collected in winter were found to be richer in oxygenated monoterpenes (68.9%), while summer ones were higher in sesquiterpene hydrocarbons (24.3%), although *O. basilicum* responded differently to temperature in terms of essential oil yield compared to *C. netepa* subsp. *spruneri*, as *O. basilicum* had the highest essential oil yield in winter (0.8%) and the lowest in summer (0.5%) [80]. Similarly to the essential oil of *C. netepa* subsp. *spruneri* outdoor and greenhouse plant *Hypericum androsaemum* essential oil had the highest level of sesquiterpene hydrocarbons in February and the lowest in September [84].

Analysis of samples from outdoor and greenhouse plants harvested in February (period when plants were in the vegetative stage) and in vitro microplants showed a similar monoterpene and sesquiterpene composition, with monoterpenes having the highest percentage (Figure 4), according to results reported for *Calamintha nepeta* plants from Tuscany Islands [59]. In Pistelli et al. [59], for experiments both in field and in vitro plants, monoterpene hydrocarbons were recorded at higher percentages to oxygenated monoterpenes, while the opposite was found in our work (Figure 4). However, as in our experiments, and also in Pistelli’s work, both field and in vitro plants had the same behavior in terms of monoterpene hydrocarbon and oxygenated monoterpene contents.

### 2.3. Chemical Composition, Seasonal Variation and Comparative Study of the Concentration and Seasonal Variation of the Main Compounds of Essential Oil Extracted from Outdoor, Greenhouse and In Vitro Plants

In the essential oil of the outdoor and greenhouse plants, 39 components were recorded, of which 35 were identified (Table 1), much more than those identified in previous studies on *C. nepeta* subsp. *spruneri* plants growing wild in various regions of Greece [36,39] (25 and 23 identified components, respectively). In the essential oil of wild *C. nepeta* subsp. *nepeta* plants in Italy, 19 to 39 components were identified in nine different populations [68], and in another study, 31 compounds in *Calamintha nepeta* plant material from Tuscany Islands [59], and 24 to 42 components were identified in plants of both subspecies from various locations in Tunisia [34].

The number of components detected in samples from in vitro plants was much lower (23) than the number of components detected in outdoor and greenhouse plants (Table 1), in agreement with corresponding research in Italy [51], where also a lower number of components (25) were detected in *C. nepeta* in vitro plants compared to outdoor plants (31).

The 39 components recorded in the samples of the outdoor and greenhouse plants, in all harvest seasons, constitute 95.4–98.8% of the total essential oil isolated from outdoor plants and 95.2–98.6% for greenhouse plants (Table 1). Regarding the 23 components detected (21 identified) in in vitro plant samples, they constitute 91.9% of the total essential oil isolated (Table 1).

**Figure 5:**
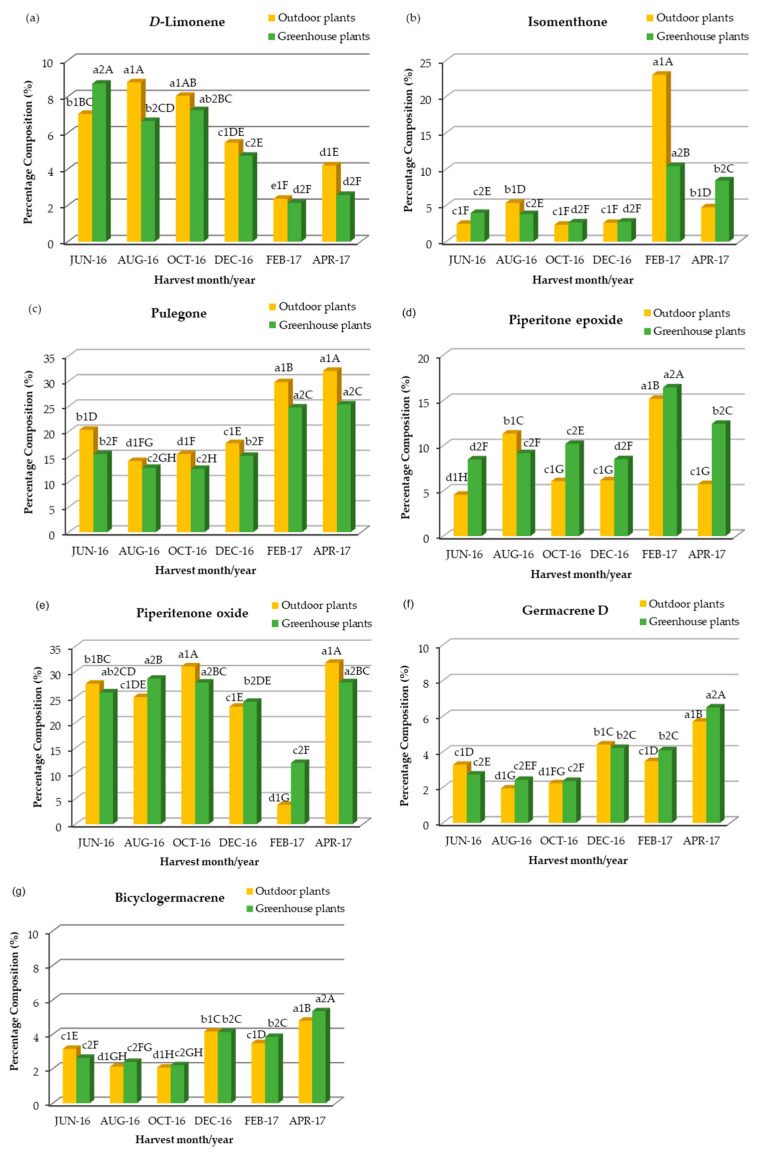
Quantitative variation in the main chemical compounds of the essential oil of *C. nepeta* subsp. *spruneri* extracted from outdoor and greenhouse plants in the months shown: (**a**) *D*-limonene, (**b**) Isomenthone, (**c**) Pulegone, (**d**) Pipetitone Epoxide, (**e**) Piperitenone Oxide, (**f**) Germacrene D, and (**g**) Bicyclogermacrene. Mean separation by Student’s *t*-test at *p* ≤ 0.05, **: significant at *p* ≤ 0.01, ***: significant at *p* ≤ 0.001, *D*-limonene: *F*_interaction_ **, F_one-way ANOVA_ ***, isomenthone: *F*_interaction_ ***, *F*_one-way ANOVA_ ***, pulegone: *F*_interaction_ ***, *F*_one-way ANOVA_ ***, pipetitone epoxide: *F* _interaction_ ***, *F*_one-way ANOVA_ ***, piperitenone oxide: *F*_interaction_ ***, *F*_one-way ANOVA_ ***, germacrene D: *F*_interaction_ ***, *F*_one-way ANOVA_ ***, bicyclogermacrene: *F*_interaction_ **, *F*_one-way ANOVA_ ***; a1, b1, c1…: comparison of means for outdoor plants (*F*: ***), a2, b2, c2…: comparison of means for greenhouse plants (*F*: ***), A, B, C…: comparison of means in both outdoor and greenhouse plants (*F*: ***).

The main chemical compounds identified in the essential oils of outdoor, greenhouse and in vitro plants were: pulegone and piperitenone oxide (13.0–32.0% and 3.8–34.2% respectively), associated with piperitone epoxide (4.6–16.4%), *D*-limonene (2.1–10.0%), isomenthone (2.3–23.0%), germacrene D (1.9–6.5%) and dicyclogermacrene (2.1–5.3%) (Table 1, Figure 5). Also, 3-octanol and carvacrol were detected at lower percentages (0.2–3.0 and 0.1–2.1%, respectively). Focusing on the percentages of the above chemical compounds in outdoor plants in June–August, in order to compare them with previous reports, where wild plants at the flowering stage (June–August) were used for essential oil extraction, we see that piperitenone oxide (27.6–25.0%) showed the highest percentages followed by pulegone (20.3–14.1%) and then *D*-limonene (7.1–8.8%), piperitone epoxide (4.6–11.3%), isomenthone (2.5–5.3%), germacrene D (3.3–1.9%) and dicyclogermacrene (3.2–2.1%). Thus, the chemotype of our plants is similar to type II of the grouping of Baldovini et al. for *C. nepeta* clones from various areas of Corsica [38], with the difference that ours do not contain menthone, but a small percentage of isomenthone. It also resembles the type I-variant (2) of the Bozovic et al. grouping [38], with the difference that, in our samples, piperitenone oxide together with piperitone epoxide were the dominant substances and not pulegone.

Previous research on wild *C. nepeta* subsp. *spruneri* plants collected in summer from various regions of Greece showed a highly variable composition of essential oils. In material on the Island of Lefkada, pulegone (39.7%), with menthone (24.7%) and isomenthone (25.6%) among others, were determined [36], similarly in material from Corinth in Peloponnese, pulegone (41%) and menthone (32%) were the major constituents along with piperitone (7.3%) and piperitenone (7%) [39], while in a report where more than 46 compounds were determined in the essential oil the major ones were found to be the two diastereoisomers of piperitone oxide (55%) and β-bisabolene (8.5%) [37]. In plants from Crete, two chemotypes were found [42], one rich in pulegone and/or menthone and/or isomenthone (the sum of these three ketones ranging from 56.8% to 89.9%) and another rich in *cis*- and *trans*-piperitone oxide and/or piperitenone oxide (the sum of these three epoxides ranging from 65.5% to 90%). Intra-populational variation has been reported for material on the Island of Zakynthos [41], where C-3 oxygenated p-menthane compounds and their precursor limonene constituted from 68.8% to 92.8% of the oils. In these samples, the main constituents of the first chemotype were pulegone, menthone, piperitenone and piperitone, while in the second chemotype *cis*- and *trans*-piperitone oxide, limonene and piperitenone oxide were found.

Thus, as in our results, in previous investigations on *C. nepeta* subsp. *spruneri* plants collected in summer from other regions of Greece [36,39,41,42], but also from Croatia [100], Tunisia [34] and Turkey [101], the chemical compounds pulegone, isomenthone, piperitone and piperitenone were determined in high percentages. However, in all the above samples, *D*-limonene, which was present in our samples, was not detected and menthone was determined as one of the main compounds, in contrast to our results, where only isomenthone was found, and at low concentrations, except for the outdoor plant harvest in February, where it was detected at a high percentage (23%). *D*-limonene was detected in *C. nepeta* subsp. *spruneri* samples from Turkey and Greece [11,69,102], while, in *Calamintha nepeta* plant material from Tuscany Islands, a high percentage of monoterpene hydrocarbons was detected, with α-pinene, β-pipene and limonene being the main representatives [59]. Regarding 3-octanol, it was detected in most previous works at low percentages (0.2–2.4%) [34,39,41,69,100,101,103,104], while carvacrol, which is the main compound in the essential oils of *Oreganum* spp. [105,106], was detected (at 0.5–5.71%) in much fewer works [59,76,107].

Regarding the comparison of the percentages of the main chemical compounds between outdoor and greenhouse plants, the two-way ANOVA revealed a significant interaction of the main experimental factors (cultivation method and harvest season), and therefore we proceeded to a comparison of the means of the experimental treatments (Table 1). In general, pulegone and *D*-limonene showed higher percentages in outdoor plants, compared to those in the greenhouse, in all sampling periods, except in June for *D*-limonene (Table 1, Figure 5). Also, isomenthone showed much higher percentages in outdoor plants in February and slightly higher in August, compared to greenhouse plants, while the opposite was observed in April and June (Table 1, Figure 5). In contrast, piperitone epoxide showed higher percentages in greenhouse plants, compared to outdoor plants, except in August (Table 1, Figure 5). Germacrene D and dicyclogermacrene had a similar behavior, i.e., in June and December they showed higher percentages in outdoor plants, compared to those in the greenhouse, while the opposite was observed in August, October, February and April (Table 1, Figure 5). Therefore, while the site of cultivation did not appear to significantly affect the yield of the essential oil (Figure 2) and its qualitative composition, it did affect its quantitative composition in some harvests depending on the harvest season. Examining the effect of harvest season on concentrations of main chemical compounds in outdoor and greenhouse plants, pulegone reached higher percentages in February (30.0% and 27.7%, respectively) and April (32.0% and 25.3%, respectively), medium percentages in June (20.3% and 15.5%, respectively) and December (17.6% and 15.1%, respectively) and lower percentages in August (14.1% and 12.7%, respectively) and October (15.5% and 12.5%, respectively) (Table 1, Figure 5).

*D*-limonene reached higher percentages in summer and autumn (June, August and October, 6.7–8.8%), medium percentages in December and April in outdoor plants and December in greenhouse plants (4.2–5.5%) and the lower percentages in February in both outdoor and greenhouse plants and in April in greenhouse plants (2.1–2.6%) (Table 1, Figure 5).

The effect of harvest season on piperitenone oxide percentage was similar to *D*-limonene, i.e., higher percentages in June, August and October (25.0–31.0%), with the difference that April percentages were similar to October, and February percentages in plants outdoors were much more reduced than in greenhouse plants (3.8 and 12.1%, respectively) (Table 1, Figure 5).

Piperitone epoxide showed the highest percentages in February in both outdoor and greenhouse plants (15.2 and 16.6%, respectively), medium percentages in all other harvest seasons in greenhouse plants (8.5–12.4%) and August in outdoor plants (11.3%) and lower percentages in all other seasons in outdoor plants (4.6–6.2%) (Table 1, Figure 5).

Isomenthone, like piperitone epoxide, showed the highest percentages in February, which were more than double in outdoor plants (23.0%) compared to greenhouse plants (10.4%). Also in April, greenhouse plants showed percentages similar to those in February (8.4%). In all other harvest months, isomenthone percentages were low (2.3–5.3%) (Table 1, Figure 5). In *Mentha* species isomethone was also found to be higher in winter than in summer [108].

Germane D and bicyclogermacrene showed a similar percentage range and values in the different harvest seasons, i.e., higher values in December, February and April (3.5–6.5%) and lower values in June, August and October (1.9–3.5%) (Table 1, Figure 5).

Seasonal variation in the percentages of the main compounds has also been observed in *Calamintha nepeta* plant material from Italy [109] and in another species of the genus, *Clinopodium macrostemum* var. *laevigatum* [110]. It has also been reported in other medicinal species of the Lamiaceae family, e.g., in *Rosmarinus officinalis* plant material from three different regions of Turkey [111], *Thymus vulgari* from Southern Brazil and New Zealand [112,113] and *Ocimum basilicum* from Pakistan [80]. As for the effect of plant growth stage on the percentages of the main compounds, pulegone and isomenthone were increased during the vegetative stage and *D*-limonene during the flowering stage (Table 1, Figure 5). However, in a wild population of *C. nepeta* subsp. *spruneri* from Zakynthos, contrary to our results, pulegone decreased in the vegetative stage, while isomenthone and *D*-limonene fluctuated similarly to our results [41]. In *C. pulegium*, pulegone was stable at the flowering and vegetative stage and decreased at the fruiting stage, where menthone was increased [55]. These inversely proportional differences in the percentage of these compounds may be due to either the different developmental stage or environmental conditions. Germane D and bicyclogermacrene percentages showed seasonal variation, in agreement with previous work on other medicinal plant species [80,84]. Various environmental parameters, such as light, temperature, CO_2_, altitude, soil water, soil fertility and salinity, influence the biosynthesis and accumulation of secondary metabolites in plant tissues and a change in an individual factor may alter the content of secondary metabolites even if other factors remain constant [45,46,47].

The chemical profile of the essential oil from the in vitro microshoots was very similar to the chemical profiles of the outdoor and greenhouse (in vivo) plants (Table 1). Piperitenone oxide, pulegone, piperitone epoxide and *D*-limonene had the highest percentages. In contrast to in vivo plants, a low percentage (0.2%) of menthone and no 3-octanol were detected (Table 1). Piperitenone oxide (34.2%) and *D*-limonene (10.0%) were at percentages similar to the June–October period in in vivo plants, pulegone (15.0%) was as in June–December in in vivo plants and piperitone epoxide (15.0%) as in February in in vivo plants. Isomenthone (4.6%) was as in June–August in in vivo plants, germacrene D (2.9%) as in June in in vivo plants and bicyclogermacrene (5.2%) as in April in in vivo plants. Furthermore, comparing the percentages of the main compounds of the essential oil extracted from in vivo cultures in February, when the plants were in the growth stage, with the oil from in vitro microshoots, we notice that pulegone and isomenthone were detected in a higher percentage in the in vivo cultures, while the opposite was observed for *D*-limonene and piperitenone oxide (Table 1). Thus, it appears that environmental parameters, mainly temperature, may be the main parameters affecting the concentrations of these compounds and not the plant growth stage (flowering or vegetative).

A previous work on *Calamintha nepeta* from Tuscany Islands, where essentials oils from in vitro plantlets were compared with those of the wild plants, found rather large, mainly quantitative, differences between them, with the largest percentage possessed by β-pinene (42.5% vs 8.4% in wild plants) and *D*-limonene (15.0% vs 34.8% in wild plants). In addition, high percentages of *cis*-muurola-3,5-diene (13.9%) and *cis*-muurola-4(14)5-diene (9.1%) were detected in samples from in vitro plants that were not found in the samples from wild plants [59].

In general, different and conflicting information is found in the literature regarding the comparison of the composition of the essential oil from in vitro and in vivo plants. The similarity of the composition has been reported in a number of medicinal species of the Lamiaceae family, e.g., *Minthostachys mollis* (Kunth) Grieseb. [114], *Origanum vulgare* ssp. *hirtum* [115], *Salvia fruticosa* [93], *Salvia sclarea* [116] and *Thymus vulgaris* [66]. On the contrary, there are comparative studies, which showed numerous differences between the two chemical profiles of the essential oil, such as for the species *Salvia przewalskii* [117] and *Eryngium planum* [95]. Light, substrate and moisture are environmental parameters that differ greatly between in vivo and in vitro culture, and thus quantitative differences in secondary metabolites are expected, as environmental variables influence the accumulation of plant secondary metabolites in plant tissues [45,46,47]. However, our work showed that micropropagation of *C. nepeta* subsp. *spruneri* by axillary shoot proliferation is a reliable method of rapid propagation of the species, leading to the production of secondary metabolites such as those found in the field-produced plants (Figure 4, Table 1). With further research, it is possible to select the most suitable chemotype by modifying the in vitro culture conditions. In vitro microshoot culture is indicated for the production of secondary metabolites, as their production has been positively correlated with cell differentiation [118]. Furthermore, microshoot cultures ensure higher genetic stability compared to callus cultures, thus allowing better standardization of secondary metabolite production. Further investigation into the use of plant growth regulators, known to favor proliferation, is likely to lead to further stimulation of terpene biosynthesis, as has been shown in other aromatic plants, which may lead to beneficial changes in the quality and quantity of the produced secondary metabolites [119]. Furthermore, in vitro cultures are offered for research and introduction to the production process of the secondary metabolites via the use of UV-B radiation, as it has been shown to affect the production of essential oil and its components in plants [120,121,122].This study showed that greenhouse cultivation as well as in vitro culture can be a valid alternative for the production of plant material of *C. nepeta* subsp. *spruneri* characterized by the same aromatic flavor and providing a similar yield of essential oil and its constituents as plants grown in the field. In addition, greenhouse and in vitro cultures can give the highest yield throughout the year if suitable ambient temperatures are provided. Furthermore, it was confirmed that the amount and quantitative composition of the essential oil depends largely on the harvest season. Therefore, for the definition of chemotypes, it is not enough to rely on a chemical analysis of an oil from a single phenophase.

## 3. Materials and Methods

### 3.1. Plant Material

#### 3.1.1. Outdoor and Greenhouse Plants (In Vivo Plants)

In the outdoor and greenhouse cultivations, six-month-old plants were used, which were grown from stem cuttings of adult wild *C. nepeta* plants, growing in Oropos, Attica (lat. 38°17′28.5″ N, long. 23°50′43.5″ E). Forty plants were grown in 10 L pots with a peat-perlite substrate (2:1 *v*/*v*), (peat (High-more with adjusted pH up to 5.5–6.5, Klasmann-Delimann Gmbh, Geeste, Germany), perlite (particles diameter 1–5 mm, Perloflor, ISOCON S.A., Athens, Greece)). The plants, until their use in the experiment, were all kept in an unheated glasshouse (lat. 37°58′57.7″ N, long. 23°42′17.2″ E), with a cooling system in the summer months and a white colored shading/thermal curtain. Shading in the greenhouse was applied between the end of April and the end of October 2016 and from the end of March to end of April 2017 (end of the experiment) at 12–5 p.m. At the beginning of January 2016, half of the plants (20 plants) were moved out of the greenhouse to adjacent outdoor benches. The same irrigation program was applied to the plants inside and outside the greenhouse, with an automatic drip irrigation system (one dropper per pot with a water supply of 4 L/h). The irrigation schedule was as follows: 24 April–25 June 2016 5 min daily, 26 April–31 October 2016 10 min daily, 1 November–15 December 2016 5 min every second day, 16 December 2016–3 March 2017 4 min every third day, 4 March–20 March 2017 4 min every second day and 21 March–30 April 2017 3 min daily.

#### 3.1.2. In Vitro Plants

The in vitro cultures were initiated from shoot tip explants excised from adult wild plants (see above) and cultured on MS medium supplemented with 1 mg L^−1^ BA [67,123]. The cultures were maintained with a number of subcultures of shoot tip and single node explants. The explants were grown in 100 mL Magenta type glass jars, which were covered with a hard plastic cap (Sigma, magenta-B caps). Each jar contained 25 mL MS medium without plant growth regulators, with 30 g L^−1^ sucrose and pH 5.7–5.8. The jars were placed in a growth chamber, at 25 ± 2 °C and a photoperiod of 16 h light of 4000 lx (37.5 μmol m^−2^ s^−1^) provided by white fluorescent lamps. Each subculture lasted 6 weeks and 40 jars (4 explants/jar) were used. The in vitro microshoots for use in the extraction of the essential oil were obtained from six subcultures.

### 3.2. Meteorological Data

**Figure 6:**
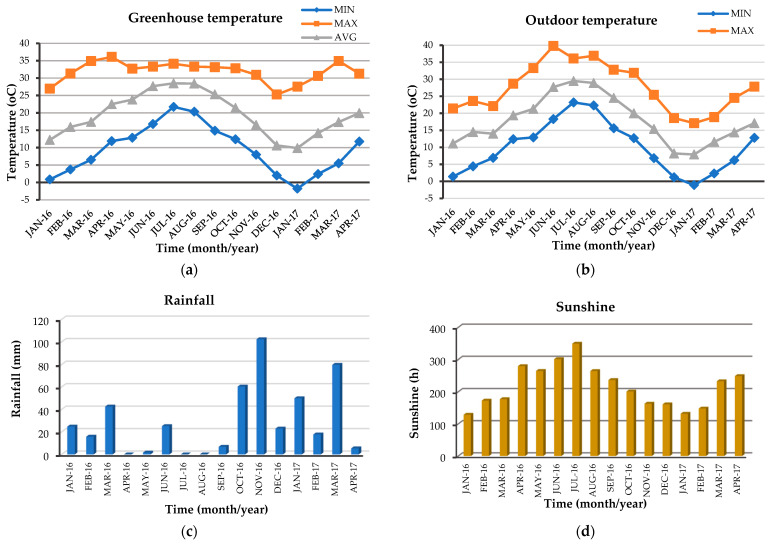
Ambient maximum, minimum and average monthly air temperature outdoors (**a**) and in the greenhouse (**b**), total monthly rainfall (**c**) and total monthly hours of sunshine (**d**) during the experimental period (23 February 2022, 11:00 p.m., meteosearch.meteo.gr/data/athens/ April–September 2021).

The ambient maximum, minimum and average monthly air temperature outdoors and in the greenhouse, total monthly rainfall and total monthly hours of sunshine during the experimental period (January 2016 to April 2017) are presented in Figure 6. These data were recorded from a meteorological cage in the proximity of the experimental plots (http://meteosearch.meteo.gr/, 23 February 2022 date of access).

### 3.3. Harvesting, Drying and Preparation of Plant Samples

#### 3.3.1. Outdoor and Greenhouse Plant Material

Harvesting of the plant material from the outdoor and greenhouse plants was done every two months, from June 2016 to April 2017, namely: June 2016, August 2016, October 2016, December 2016, February 2017 and April 2017. Morning hours, the above-ground part of each plant was cut 10 cm above the pot rim. Shoots were transferred to black nylon bags and taken to a dark room, weighed on a precision balance (Mettler Toledo PJ3600/PJ 3600 DeltaRange Precision Balance/Scale, 0.01 g to 3200 g) and spread on a bench to allow natural drying (room temperature, shade). Every three days they were weighed and, when no further reduction in weight was observed, the leaves (or the leaves and flowers at times when the plants were in bloom) were removed from the stems and stored in tightly closed nylon bags, at room temperature and shaded. Flowers were present in the June, August, October and December harvests.

#### 3.3.2. In Vitro Plant Material

After the end of each in vitro subculture, microshoots (2.7 cm length and 4 nodes, on average) were collected (Figure 1E), naturally dried and stored as described for the outdoor and greenhouse plant material, except that the leaves were not separated from the stems.

### 3.4. Essential Oil Isolation

The isolation of essential oil from the dried plant material of outdoor and greenhouse plants and its analysis were done separately for each harvest period, within 1–2 months of harvest, while the isolation of essential oil from in vitro plant material was done after pooling samples of the six subcultures. The essential oil was obtained by the method of hydrodistillation in a Clevenger apparatus. Samples (30 g for outdoor and greenhouse plant material and 15 g for in vitro plant material) were ground and subjected to hydrodistillation until the layer of distilled essential oil did not rise any further (duration 3 h). The ratio of plant material to water in the boiling flask was 1:15.

The essential oils were collected in sealed glass vessels and stored at −18 °C until their analysis by gas chromatography-mass spectrometry (GC-MS), which took place about 15 d after essential oil isolation.

### 3.5. Gas Chromatography-Mass Spectrometry (GC/MS) Analysis

The qualitative analysis of the essential oil was performed using a gas chromatograph (GC) (Hewlett Packard 5890 II), equipped with an Rtx-5MS capillary column (30 m × 0.25 mm, film thickness 0.25 μm) and a Hewlett Packard 5972 (70 eV) mass selective detector. Helium was used as carrier gas at a flow rate of 1 mL min^–1^. The temperature at the injector was 220 °C and at the MS transfer line was 250 °C. The initial temperature of the column was 60 °C and then increased gradually to 210 °C at the rate of 3 °C min^–1^. The analysis program had a total duration of 50 min. The volume of the sample to be analyzed was 1 μL (splitless mode). Essential oil samples for analysis were diluted 1/100 (*v*/*v*) with 99.8% pure acetone (GC-MS analytical grade). Three replicates were performed in each sampling period of the outdoor and greenhouse plant material, as well as of the plant material from in vitro culture.

Component identification was based on comparison of their relative retention indices and mass spectra with those of the Nist 98 and Wiley 275 MS libraries, with GC-MS system data and literature data [124].

A solution of n-alkanes (C_8_–C_24_) was used for the determination of the Retention Index (RI). The RI of each compound calculated by the following formula [125]:

$$RI=\frac{100\times n+[\log\left(RTunkown-v\right)-log(RTsmaller\;alkane-v)]}{\log\left(RTlarger\;alkane-v\right)-log(RTsmaller\;alkane-v)}$$

where: n = the number of C in the smaller alkane; RT*unknown* = the retention time of the unknown in seconds; *v* = column void time; RT*smaller alkane* = the retention time of the smaller alkane; and RT*larger alkane* = the retention time of the larger alkane.

The percentages of the compounds were obtained automatically based on peak area data.

### 3.6. Statistical Analysis

A completely randomized design was used. The significance of the results was tested by either one- or two-way analysis of variance (ANOVA) and the means of the treatments were compared by Student’s *t*-test at *p* ≤ 0.05 (JMP 11.0 software, SAS Institute Inc., Cary, NC, 2013, USA).

The following statistical analyses were performed: (A) one-way ANOVA to compare the percentages at different harvest dates (harvest seasons) of (a) essential oil, (b) each chemical compound and (c) each chemical group of the (i) outdoor plants and (ii) greenhouse plants; (B) two-way ANOVA with the cultivation method (outdoor, greenhouse) and the harvest season as main experimental factors, to compare the percentage of (a) essential oil, (b) each chemical compound and (c) each chemical group.

## 4. Conclusions

*Clinopodium nepeta* subsp. *spruneri* plants yielded a similar essential oil in outdoor (0.9–2.5%), greenhouse (1.2–2.5%) and in vitro (1.7%) cultivation.

Essential oil yield varied similarly in outdoor and greenhouse plants throughout the year, reaching higher percentages in the warm/hot harvest seasons, i.e., April (plants in vegetative stage) and June to October (plants in flowering stage) (2.1–2.6%), compared to the cool harvesting seasons of December (plants in flowering stage) (1.5%) and February (plants in vegetative stage) (0.9%).

In the oil of outdoor and greenhouse plants, more chemical compounds were identified compared to those of in vitro plants (35 vs. 21, respectively), but the main compounds were the same, i.e., pulegone, piperitenone oxide, piperitone epoxide, D-limonene, isomenthone, germacrene D and dicyclogermacrene.

In outdoor and greenhouse cultivations, the harvest season affected the percentage composition of the oil, and this was mainly attributed to the ambient temperatures.

Greenhouse cultivation as well as in vitro culture were equally efficient in yielding essential oil and its components as the outdoor cultivation, being a valid alternative for the production of plant material of *C. nepeta* subsp. *spruneri* regardless of geographical and seasonal parameters, providing the highest yield throughout the year, as long as suitable environmental conditions are provided.

Further research may lead to the selection of more suitable chemotypes by modifying the conditions in the greenhouse, and especially in vitro. The use of plant growth regulators, modification of temperature, as well as modification of intensity, quality and duration of lighting can modify the composition and quantity of secondary metabolites in the essential oil of *C. nepeta* subsp. *spruneri.*

## Figures and Tables

**Table 1:** Seasonal variation in the content (%) and chemical composition of essential oil extracted from outdoor, greenhouse and in vitro cultured *C. nepeta* subsp. *nepeta* plants by hydrodistillation and determined by GC/MS in the indicated months. Compounds are listed in the order of elution from an Rtx-5MS capillary column.

			June	August	October	December	February	April	June	August	October	December	February	April	
	R.I.exp	Compounds	Outdoor Plants						Greenhouse Plants						In Vitro Plants
1.	938	α-Pinene	0.2	0.6	0.6	0.6	0.2	0.2	0.6	0.4	0.7	0.5	0.5	0.2	0.2
2.	981	*β*-Pinene	0.4	0.6	0.6	0.6	0.3	0.4	0.7	0.5	0.7	0.5	0.8	0.4	0.2
3.	990	*β*-Myrcene	0.3	0.5	0.3	0.4	0.1	0.1	0.4	0.4	0.5	0.4	0.3	0.1	0.3
4.	994	3-Octanol	2.5	2.9	3.0	2.7	1.0	1.2	2.9	2.6	2.6	2.5	1.8	1.0	0.2
5.	1032	***D*-Limonene**	**7.1**	**8.8**	**8.0**	**5.5**	**2.4**	**4.2**	**8.7**	**6.7**	**7.2**	**4.7**	**2.1**	**2.6**	**10.0**
6.	1062	*γ*-Terpinene	nd	0.2	0.1	0.1	nd	nd	0.1	0.1	0.1	0.1	nd	nd	-
7.	1095	3-Nonanol	0.2	0.6	0.3	0.2	nd	nd	0.3	0.4	0.3	0.2	0.3	0.2	-
8.	1099	*trans*-Sabinene hydrate	0.1	nd	0.1	0.1	nd	nd	0.1	nd	nd	nd	nd	nd	-
9.	1102	*n*-Nonanal	0.1	0.4	0.1	0.1	1.0	0.3	0.2	0.2	0.1	0.1	nd	nd	0.1
10.	1156	**Menthone**	**-**	**-**	**-**	**-**	**-**	**-**	**-**	**-**	**-**	**-**	**-**	**-**	**0.2**
11.	1165	**Isomenthone**	**2.5**	**5.3**	**2.3**	**2.6**	**23.0**	**4.7**	**4.0**	**3.8**	**2.6**	**2.7**	**10.4**	**8.4**	**4.6**
12.	1178	Isopulegone *	0.6	0.3	0.3	1.1	0.9	0.4	0.3	0.2	nd	1.1	1.1	0.4	0.1
13.	1182	Unknown (M^1^) **	0.5	0.8	0.4	0.3	0.7	nd	0.4	0.6	0.8	0.2	nd	nd	-
14.	1205	Unknown (M^2^) **	0.3	0.9	0.5	0.5	1.3	nd	0.7	0.8	0.9	0.7	0.7	nd	-
15.	1225	2-Ethenyl-1-methoxy-3-methylbenzene	1.0	0.8	1.1	0.9	0.1	0.1	1.0	1.2	1.1	1.0	1.0	0.1	-
16.	1240	**Pulegone**	**20.3**	**14.1**	**15.5**	**17.6**	**29.8**	**32.0**	**15.5**	**12.7**	**12.5**	**15.1**	**24.7**	**25.3**	**15.0**
17.	1256	Piperitone epoxide *	**4.6**	**11.3**	**6.1**	**6.2**	**15.2**	**5.7**	**8.5**	**9.2**	**10.2**	**8.5**	**16.4**	**12.4**	**15.5**
18.	1275	Isopiperitenone	0.9	0.9	1.0	0.6	0.5	0.5	1.0	1.0	0.9	0.8	0.6	0.5	0.3
19.	1293	Unknown (M^3^) **	8.8	7.2	9.9	7.1	1.3	0.2	8.6	9.7	9.1	7.7	4.0	0.2	0.2
20.	1298	Thymol	0.6	1.0	0.7	0.8	1.0	0.3	0.8	0.9	1.0	0.9	1.2	0.3	0.2
21.	1301	2-Hydroxypiperitone	0.3	0.9	0.4	0.5	1.5	nd	0.8	0.9	1.0	0.8	1.8	nd	
22.	1309	Carvacrol	0.5	0.1	0.1	0.4	0.3	nd	0.3	0.1	2.1	0.6	0.5	0.1	0.5
23.	1343	Piperitenone	1.2	0.7	1.0	1.9	1.1	1.3	1.2	0.8	2.1	1.3	1.2	1.4	1.0
24.	1366	**Piperitenone oxide**	**27.6**	**25.0**	**31.0**	**23.2**	**3.8**	**31.8**	**25.9**	**28.6**	**27.9**	**24.1**	**12.1**	**27.9**	**34.2**
25.	1383	*α*-Copaene	0.2	0.2	0.3	0.5	0.1	0.3	0.3	0.3	0.3	0.4	0.2	0.2	-
26.	1392	*β*-Bourbonene	0.7	0.3	0.8	0.3	0.2	0.2	0.6	0.8	0.7	0.8	0.2	nd	-
27.	1397	*β*-Elemene	0.3	0.2	0.2	0.4	0.2	0.2	0.2	0.2	0.2	0.4	0.3	0.2	0.1
28.	1407	Unknown (M^4^) **	5.8	4.6	6.3	5.2	0.5	0.4	5.7	6.4	6.0	5.5	2.4	0.4	0.3
29.	1428	*β*-Caryuphyllene	2.6	1.6	1.8	3.9	3.8	3.5	2.0	1.7	1.5	3.2	3.7	3.6	0.5
30.	1436	*β*-Copaene	0.2	0.1	0.2	0.4	0.2	nd	0.2	0.1	0.1	0.3	0.3	nd	-
31.	1447	Aromadendrene	0.2	0.1	0.1	0.3	0.1	nd	0.1	0.2	0.2	0.4	0.3	nd	-
32.	1461	*α*-Caryoplyllene	0.1	0.1	0.2	0.4	0.2	0.2	0.2	0.2	0.2	0.3	0.3	0.2	-
33.	1470	*cis*-Muurola-4(14)5-diene	0.2	0.1	0.1	0.3	0.2	0.1	0.2	0.1	0.1	0.2	0.2	0.1	0.1
34.	1483	*γ*-Muurolene	0.2	0.1	0.1	0.3	0.2	nd	0.2	0.1	0.1	0.2	0.2	nd	-
35.	1489	**Germacrene D**	**3.3**	**1.9**	**2.2**	**4.4**	**3.5**	**5.7**	**2.7**	**2.4**	**2.4**	**4.2**	**4.1**	**6.5**	**2.9**
36.	1504	**Bicyclogermacrene**	**3.2**	**2.1**	**2.1**	**4.2**	**3.5**	**4.8**	**2.6**	**2.4**	**2.2**	**4.1**	**3.9**	**5.3**	**5.2**
37.	1520	*γ*-Cadinene	0.2	0.1	0.1	0.3	0.1	nd	0.1	0.1	0.1	0.2	0.2	nd	-
38.	1528	*δ*-Cadinene	0.2	0.1	0.1	0.3	0.2	nd	0.2	0.2	0.1	0.3	0.3	0.1	-
39.	1544	*α*-Cadinene	tr	nd	nd	0.1	nd	nd	0.1	tr	nd	0.1	0.1	nd	-
40.	1591	Spathulenol	0.1	tr	0.2	0.1	nd	nd	0.1	0.1	nd	0.1	nd	nd	-

R.I.exp. Experimental retention index. * Correct isomer not identified. ** Main qualifying ions (*m*/*z*) and their relative abundance (%). Unknown (M^1^): 139 (100), 55 (62), 69 (61), 97 (59) **. Unknown (M^2^): 139 (100), 97 (67), 69 (64), 55 (61) **. Unknown (M^3^): 138 (100), 137 (91), 68 (96), 67 (89), 123 (43), 166 (6,5) **. Unknown (M^4^): 166 (100), 123 (60), 105 (22), 137 (19), 69 (18) **. tr, trace (<0.1%), nd: not detected. Bold indicate the main chemical compounds of the essential oil.

## Data Availability

Data are available upon request.

## References

[1] Adams M., Berset C., Kessler M., Hamburger M. (2009). Medicinal herbs for the treatment of rheumatic disorders—A survey of European herbals from the 16th and 17th century. J. Ethnopharmacol..

[2] Nacer-bey N., Chabane D., Haouli A.K., Aribi I. (2015). Traditional herbal medicine in Jijel region, Northeast of Algeria. Adv. Environ. Biol..

[3] Rashrash M., Schommer J.C., Brown L.M. (2017). Prevalence and Predictors of Herbal Medicine Use Among Adults in the United States. J. Patient Exp..

[4] Salim M.A., Ranjitkar S., Hart R., Khan T., Ali S., Kiran C., Parveen A., Batool Z., Bano S., Xu J. (2019). Regional trade of medicinal plants has facilitated the retention of traditional knowledge: Case study in Gilgit-Baltistan Pakistan. J. Ethnobiol. Ethnomed..

[5] Zhang L., Zhuang H., Zhang Y., Wang L., Zhang Y., Geng Y., Yi G., Pei S., Wang Y. (2018). Plants for health: An ethnobotanical 25-year repeat survey of traditional medicine sold in a major marketplace in North-west Yunnan, China. J. Ethnopharmacol..

[6] Alotiby A.A., Al-Harbi L.N. (2021). Prevalence of using herbs and natural products as a protective measure during the COVID-19 pandemic among the Saudi population: An online cross-sectional survey. Saudi Pharm. J..

[7] Ball P.W., Getliffe F., Tutin T., Heywood V., Burges N., Moore D., Valentine D., Walters S., Webb D. (1968). Calamintha Miller. Flora Europaea.

[8] Davis P.H., Leblebici E. (1987). Flora of Turkey and East Aegean Islands.

[9] Davis P.H., Mill R.R., Tan K. (1988). Flora of Turkey and East Aegean Islands.

[10] Alan S., Ocak A. (2009). Taxonomical and morphological studies on the genus *Calamintha* Miller (Lamiaceae) in Turkey. Biol. Divers. Conserv..

[11] Bown D. (1995). The Herb Society of America Encyclopedia of Herbs and Their Uses.

[12] Chevallier A. (2001). Encyclopedia of Medicinal Plants.

[13] Brankovic S.V., Kitic D.V., Radenkovic M.M., Veljkovic S.M., Golubovic T.D. (2009). Calcium blocking activity as a mechanism of the spasmolytic effect of the essential oil of *Calamintha glandulosa* Silic on the isolated rat ileum. Gen. Physiol. Biophys..

[14] Flamini G., Cioni P.L., Puleio R., Morelli I., Panizzi L. (1999). Antimicrobial activity of the essential oil of *Calamintha nepeta* and its constituent pulegone against bacteria and fungi. Phyther. Res..

[15] Monforte M.T., Tzakou O., Nostro A., Zimbalatti V., Galati E.M. (2011). Chemical composition and biological activities of *Calamintha officinalis* Moench essential oil. J. Med. Food.

[16] Pardo-de-Santayana M., Tardío J., Blanco E., Carvalho A., Lastra J., San Miguel E., Morales R. (2007). Traditional knowledge of wild edible plants used in the northwest of the Iberian Peninsula (Spain and Portugal): A comparative study. J. Ethnobiol. Ethnomed..

[17] Sarac N., Ugur A. (2009). The in vitro antimicrobial activities of the essential oils of some Lamiaceae species from Turkey. J. Med. Food.

[18] Verma D., Irchhaiya M., Singh R., Kailasiya P.P., Kanaujia V. (2011). Studies on antiulcer activity of essential oil of *Calamintha officinalis* Moench. Int. J. Res. Pharm. Sci..

[19] Amira S., Dade M., Schinella G., Ríos J.L. (2012). Anti-inflammatory, anti-oxidant, and apoptotic activities of four plant species used in folk medicine in the Mediterranean basin. Pak. J. Pharm. Sci..

[20] Ceker S., Agar G., Alpsoy L., Nardemir G., Kizil H.E. (2013). Protective role of essential oils of *Calamintha nepeta* L. on oxidative and genotoxic damage caused by Alfatoxin B1 in vitro. Fresenius Environ. Bull..

[21] Sarikurkcu C., Ozer M.S., Tepe B., Dilek E., Ceylan O. (2015). Phenolic composition, antioxidant and enzyme inhibitory activities of acetone, methanol and water extracts of *Clinopodium vulgare* L. subsp. vulgare L. Ind. Crops Prod..

[22] Shams Moattar F., Sariri R., Giahi M., Yaghmaee P. (2018). Essential oil composition and antioxidant activity of *Calamintha officinalis* Moench. J. Appl. Biotechnol. Rep..

[23] Beddiar H., Boudiba S., Benahmed M., Tamfu A.N., Ceylan Ö., Hanini K., Küçükaydın S., Elomari A., Chawki B., Laouer H. (2021). Chemical Composition, Anti-Quorum Sensing, Enzyme Inhibitory, and Antioxidant Properties of Phenolic Extracts of *Clinopodium nepeta* L. Kuntze. Plants.

[24] Petrova M., Dimitrova L., Dimitrova M., Denev P., Teneva D., Georgieva A., Petkova-Kirova P., Lazarova M., Tasheva K. (2023). Antitumor and Antioxidant Activities of In Vitro Cultivated and Wild-Growing *Clinopodium vulgare* L. Plants. Plants.

[25] Rodenak-Kladniew B., Castro M.A., Gambaro R.C., Girotti J., Cisneros J.S., Viña S., Padula G., Crespo R., Castro G.R., Gehring S. (2023). Cytotoxic Screening and Enhanced Anticancer Activity of *Lippia alba* and *Clinopodium nepeta* Essential Oils-Loaded Biocompatible Lipid Nanoparticles against Lung and Colon Cancer Cells. Pharmaceutics.

[26] Pignatti S. (1982). Flora D’Italia.

[27] Filibeck G., Cornelini P., Petrella P. (2012). Floristic analysis of a high-speed railway embankment in a Mediterranean landscape. Acta Bot. Croat..

[28] Dimopoulos P., Raus T., Bergmeier E., Constantinidis T., Iatrou G., Kokkini S., Strid A., Tzanoudakis D. (2013). Vascular Plants of Greece: An Annotated Checklist.

[29] Brussell D. (2004). Medicinal Plants of Mt. Pelion, Greece. Econ. Bot..

[30] Baytop T. (1999). Therapy with Medicinal Plants in Turkey Past and Present.

[31] Marongiu B., Piras A., Porcedda S., Falconieri D., Maxia A., Gonçalves M.J., Cavaleiro C., Salgueiro L. (2010). Chemical composition and biological assays of essential oils of *Calamintha nepeta* (L.) Savi subsp. *nepeta* (Lamiaceae). Nat. Prod. Res..

[32] Demirci B., Temel H.E., Portakal T., Kırmızıbekmez H., Demirci F., Baser K.H.C. (2011). Inhibitory effect of *Calamintha nepeta* subsp. *glandulosa* essential oil on lipoxygenase. Turk. J. Biochem..

[33] Božović M., Ragno R. (2017). *Calamintha nepeta* (L.) Savi and its Main Essential Oil Constituent Pulegone: Biological Activities and Chemistry—A review. Molecules.

[34] Debbabi H., Mokni R.E., Chaieb I., Nardoni S., Maggi F., Caprioli G., Hammami S. (2020). Chemical Composition, Antifungal and Insecticidal Activities of the Essential Oils from Tunisian *Clinopodium nepeta* subsp. *nepeta* and *Clinopodium nepeta* subsp. *glandulosum*. Molecules.

[35] Lee S., Tsao R., Peterson C., Coats J.R. (1997). Insecticidal activity of monoterpenoids to western corn rootworm (Coleoptera: Chrysomelidae), twospotted spider mite (Acari: Tetranychidae), and house fly (Diptera: Muscidae). J. Econ. Entomol..

[36] Souleles C., Argyriadou N., Philianos S. (1987). Constituents of the essential oil of *Calamintha nepeta*. J. Nat. Prod..

[37] Kokkalou E., Stefanou E. (1990). The volatile oil of *Calamintha nepeta* (L.) Savi ssp. *glandulosa* (Req.) Ball, endemic to Greece. Flavour Fragr. J..

[38] Baldovini N., Ristorcelli D., Tomi F., Casanova J. (2000). Intraspecific variability of the essential oil of *Calamintha nepeta* from Corsica (France). Flavour Fragr. J..

[39] Couladis M., Tzakou O. (2001). Essential oil of *Calamintha nepeta* subsp. *glandulosa* from Greece. J. Essent. Oil Res..

[40] Kitic D., Stojanović G., Palic R., Randjelovic V. (2005). Chemical composition and microbial activity of the essential oil of *Calamintha nepeta* (L.) Savi ssp. *nepeta* var. *subisodonda* (Borb.) Hayek from Serbia. J. Essent. Oil Res..

[41] Cook C.M., Lanaras T., Kokkini S. (2007). Essential oils of two *Calamintha glandulosa* (Req.) Bentham chemotypes in a wild population from Zakynthos, Greece. J. Essent. Oil Res..

[42] Karousou R., Hanlidou E., Lazari D. (2012). Essential-oil diversity of three *Calamintha* species from Greece. Chem. Biodivers..

[43] Baser K.H.C., Kirimer N., Tümen G. (1998). Pulegone-rich essential oils of Turkey. J. Essent. Oil Res..

[44] Dhingra A.K., Chopra B., Bhardwaj S., Dhar K.L. (2011). Synthesis and characterization of novel pulegone derivatives as substitutes of 4-(1,1 dimethylethyl) cyclohexan-1-ol acetate. J. Pharm. Res..

[45] Yang L., Kui-Shan Wen K.-S., Ruan X., Zhao Y.-X., Wei F., Wang Q. (2018). Response of Plant Secondary Metabolites to Environmental Factors. Molecules.

[46] Jan R., Asaf S., Numan M., Lubna, Kim K.-M. (2021). Plant Secondary Metabolite Biosynthesis and Transcriptional Regulation in Response to Biotic and Abiotic Stress Conditions. Agronomy.

[47] Pant P., Pandey S., Dall’Acqua S. (2021). The Influence of Environmental Conditions on Secondary Metabolites in Medicinal Plants: A Literature Review. Chem. Biodivers..

[48] Giuffrè A.M., Nobile R. (2020). *Citrus bergamia*, Risso: The peel, the juice and the seed oil of the bergamot fruit of Reggio Calabria (South Italy). Emir. J. Food Agric..

[49] Şanli A., Karadoğan T. (2017). Geographical impact on essential oil composition of endemic *Kundmannia anatolica* Hub.-Mor. (Apiaceae). Afr. J. Tradit. Complement. Altern. Med..

[50] Gioffrè G., Ursino D., Labate M.L.C., Giuffrè A.M. (2020). The peel essential oil composition of bergamot fruit (*Citrus bergamia*, Risso) of Reggio Calabria (Italy): A review. Emir. J. Food Agric..

[51] de Araujo J., Silvestre W.P., Pauletti G.F., Muniz L.A.R. (2023). Influence of the Absolute Pressure of the Extraction System on the Yield and Composition of *Corymbia citriodora* (Hook.) K.D. Hill and L.A.S. Johnson Leaf Essential Oil Extracted by Steam Distillation. ChemEngineering.

[52] Perry N.B., Anderson R.E., Brennan N.J., Douglas M.H., Heaney A.J., McGimpsey J.A., Smallfield B.M. (1999). Essential oils from dalmatian sage (*Salvia officinalis* L.): Variations among individuals, plant parts, seasons, and sites. J. Agric. Food Chem..

[53] Yavari A., Nazeri V., Sefidkon F., Hassani M.E. (2010). Influence of some environmental factors on the essential oil variability of *Thymus migricus*. Nat. Prod. Commun..

[54] Fernández-Sestelo M., Carrillo J.M. (2020). Environmental effects on yield and composition of essential oil in wild populations of spike lavender (*Lavandula latifolia* Medik.). Agriculture.

[55] Slavkovska V., Zlatković B., Bräuchler C., Stojanović D., Tzakou O., Couladis M. (2013). Variations of essential oil characteristics of *Clinopodium pulegium* (Lamiaceae) depending on phenological stage. Bot. Serbica.

[56] Matkowski A. (2008). Plant in vitro culture for the production of antioxidants—A review. Biotechnol. Adv..

[57] Silva S., Sato A., Lage C.L.S., San Gil R.A.S., de Azevedo D., Esquibel M.A. (2005). Essential oil composition of *Mellisa officinalis* L. in vitro produced under the influence of growth regulators. J. Braz. Chem. Soc..

[58] Affonso V.R., Bizzo H.R., Lima S.S., Esquibel M.A., Sato A. (2007). Solid phase microextraction (SPME) analysis of volatile compounds produced by in vitro shoots of *Lantana camara* L. under the influence of auxins and cytokinins. J. Braz. Chem. Soc..

[59] Pistelli L., Noccioli C., D’Angiolillo F., Pistelli L. (2013). Composition of volatile in micropropagated and field grown aromatic plants from Tuscany Islands. Acta Biochim. Pol..

[60] Vázquez A.M., Goleniowski M.E., Aimar M.L., Diaz M.S., Demmel G.I., Decarlini M.F., Cantero J.J. (2018). Profile Characterization of Volatile Organic Compounds on in vitro Propagated Plants of *Clinopodium odorum* and it’s Comparison with the Wild Plant. SAJ Pharm. Pharmacol..

[61] Lila M.A., Trigiano R.N., Gray D. (2005). Valuable Secondary Products from In Vitro Culture. Plant Development and Biotechnology.

[62] Gounaris Y. (2010). Biotechnology for the production of essential oils, flavours and volatile isolates. A review. Flavour Fragr. J..

[63] Collin H.A. (2001). Secondary product formation in plant tissue cultures. Plant Growth Regul..

[64] Amaral C.L.F., Silva A.B. (2003). Melhoramento biotecnológico de plantas medicinais. Biotecnol. Ciência Desenvolv..

[65] Wawrosch C., Zotchev S.B. (2021). Production of bioactive plant secondary metabolites through in vitro technologies—Status and outlook. Appl. Microbiol. Biotechnol..

[66] Affonso V.R., Bizzo H.R., Lage C.L.S., Sato A. (2009). Influence of growth regulators in biomass production and volatile profile of in vitro plantlets of *Thymus vulgaris* (L.). J. Agric. Food Chem..

[67] Vlachou G., Papafotiou M., Bertsouklis K.F. (2016). In vitro propagation of *Calamintha nepeta*. Acta Hortic..

[68] Vlachou G., Papafotiou M., Bertsouklis K. (2019). Studies on seed germination and micropropagation of *Clinopodium nepeta*: A medicinal and aromatic plant. Hort. Sci..

[69] Şarer E., Solakel Pançalı S. (1998). Composition of the essential oil from *Calamintha nepeta* (L.) Savi ssp. *glandulosa* (Req.) PW Ball. Flavour Fragr. J..

[70] Bellomaria B., Valentini G. (1985). Composizione dell’olio essenziale di *Calamintha nepeta* subsp. *glandulosa*. Plant Biosyst..

[71] De Pooter H.L., de Buyck L.F., Schamp N.M. (1986). The volatiles of *Calamintha nepeta* subsp. *glandusola*. Phytochemistry.

[72] Akgül A., De Pooter H.L., De Buyck L.F. (1991). The essential oils of *Calamintha nepeta* subsp. *glandulosa* and *Ziziphora clinopodioides* from Turkey. J. Essent. Oil Res..

[73] Panizzi L., Flamini G., Cioni P.L., Morelli I. (1993). Composition and antimicrobial properties of essential oils of four Mediterranean Lamiaceae. J. Ethnopharmacol..

[74] Mastelić J., Miloš M., Kuštrak D., Radonić A. (1998). The essential oil and glycosidically bound volatile compounds of *Calamintha nepeta* (L.) Savi. Croat. Chem. Acta.

[75] Kitic D., Jovanovic T., Ristic M., Palic R., Stojanovic G. (2002). Chemical composition and antimicrobial activity of the essential oil of *Calamintha nepeta* (L.) Savi ssp. *glandulosa* (Req.) PW Ball from Montenegro. J. Essent. Oil Res..

[76] Hodaj-Çeliku E., Tsiftsoglou O., Shuka L., Abazi S., Hadjipavlou-Litina D., Lazari D. (2017). Antioxidant activity and chemical composition of essential oils of some aromatic and medicinal plants from Albania. Nat. Prod. Commun..

[77] Arantes S.M., Piçarra A., Guerreiro M., Salvador C., Candeias F., Caldeira A.T., Martins M.R. (2019). Toxicological and pharmacological properties of essential oils of *Calamintha nepeta*, *Origanum virens* and *Thymus mastichina* of Alentejo (Portugal). Food Chem. Toxicol..

[78] Negro C., Notarnicola S., De Bellis L., Miceli A. (2013). Intraspecific variability of the essential oil of *Calamintha nepeta* subsp. *nepeta* from Southern Italy (Apulia). Nat. Prod. Res..

[79] Kofidis G., Bosabalidis A., Kokkini S. (2004). Seasonal variation of essential oils in a linalool-rich chemotype of *Mentha spicata* grown wild in Greece. J. Essent. Oil Res..

[80] Hussain A.I., Anwar F., Sherazi S.T.H., Przybylski R. (2008). Chemical composition, antioxidant and antimicrobial activities of basil (*Ocimum basilicum*) essential oils depends on seasonal variations. Food Chem..

[81] Khalid A.K., El-Gohary A.E. (2014). Effect of seasonal variations on essential oil production and composition of *Plectranthus amboinicus* (Lour.) grow in Egypt. Int. Food Res. J..

[82] Lakušić B., Ristić M., Slavkovska V., Lakušić D. (2013). Seasonal variations in the composition of the essential oils of rosemary (*Rosmarinus officinalis*, Lamiaceae). Nat. Prod. Commun..

[83] Melito S., Petretto G.L., Chahine S., Pintore G., Chessa M. (2019). Seasonal variation of essential oil in *Rosmarinus officinalis* leaves in Sardinia. Nat. Prod. Commun..

[84] Guedes A.P., Amorim L.R., Vicente A., Fernandes-Ferreira M. (2004). Variation of the essential oil content and composition in leaves from cultivated plants of *Hypericum androsaemum* L.. Phytochem. Anal. Int. J. Plant Chem. Biochem. Tech..

[85] Lakušić B., Ristić M., Slavkovska V., Stojanović D., Lakušić D. (2013). Variations in essential oil yields and compositions of *Salvia officinalis* (Lamiaceae) at different developmental stages. Bot. Serbica.

[86] Chericoni S., Flamini G., Campeol E., Cioni P.L., Morelli I. (2004). GC–MS analyses of the essential oil from the aerial parts of *Artemisia verlotiorum*: Variability during the year. Biochem. Syst. Ecol..

[87] Palá-Paúl J., Pérez-Alonso M.J., Velasco-Negueruela A., Palá-Paúl R., Sanz J., Conejero F. (2001). Seasonal variation in chemical constituents of *Santolina rosmarinifolia* L. ssp. *rosmarinifolia*. Biochem. Syst. Ecol..

[88] Karousou R., Kokkini S., Bessière J.M., Vokou D. (1996). *Calamintha cretica* (Lamiaceae), a Cretan endemic: Distribution and essential oil composition. Nord. J. Bot..

[89] Juliani H.R., Koroch A.R., Juliani H.R., Trippi V.S., Zygadlo J.A. (2002). Intraespecific variation in leaf oils of *Lippia junelliana* (mold.) tronc. Biochem. Syst. Ecol..

[90] Aziz E.E., Badawy E.M., Zheljazkov V.D., Nicola S.M., Fouad H. (2019). Yield and chemical composition of essential oil of *Achillea millefolium* L. as affected by harvest time. Egypt. J. Chem..

[91] Gazim Z.C., Amorim A.C.L., Hovell A.M.C., Rezende C.M., Nascimento I.A., Ferreira G.A., Cortez D.A.G. (2010). Seasonal variation, chemical composition, and analgesic and antimicrobial activities of the essential oil from leaves of *Tetradenia riparia* (Hochst.) Codd in Southern Brazil. Molecules.

[92] Hirata T., Murakami S., Ogihara K., Suga T. (1990). Volatile monoterpenoid constituents of the plantlets of *Mentha spicata* produced by shoot tip culture. Phytochemistry.

[93] Arikat N.A., Jawad F.M., Karam N.S., Shibli R.A. (2004). Micropropagation and accumulation of essential oils in wild sage (*Salvia fruticosa* Mill.). Sci. Hortic..

[94] Amaral-Baroli A., Lago J.H.G., de Almeida C.V., de Almeida M., Scotti M.T., Leone G.F., Soares M.G., Cavalari A.A., Sartorelli P. (2016). Variability in essential oil composition produced by micropropagated (in vitro), acclimated (ex vitro) and in-field plants of *Ocimum basilicum* (Lamiaceae). Ind. Crops Prod..

[95] Thiem B., Kikowska M., Kurowska A., Kalemba D. (2011). Essential oil composition of the different parts and in vitro shoot culture of *Eryngium planum* L.. Molecules.

[96] Nogueira J.M.F., Romano A. (2002). Essential oils from micropropagated plants of *Lavandula viridis*. Phytochem. Anal. Int. J. Plant Chem. Biochem. Tech..

[97] Manan A.A., Taha R.M., Mubarak E.E., Elias H. (2016). In vitro flowering, glandular trichomes ultrastructure, and essential oil accumulation in micropropagated *Ocimum basilicum* L.. Vitr. Cell. Dev. Biol.-Plant..

[98] Shen X., Kane M.E., Chen J. (2008). Effects of genotype, explant source, and plant growth regulators on indirect shoot organogenesis in *Dieffenbachia* cultivars. Vitr. Cell. Dev. Biol.-Plant.

[99] Amoo S., Adeyemi A., Staden J. (2013). Shoot proliferation and rooting treatments influence secondary metabolite production and antioxidant activity in tissue culture-derived *Aloe arborescens* grown ex vitro. Plant Growth Regul..

[100] Ćavar S., Vidic D., Maksimović M. (2012). Volatile constituents, phenolic compounds, and antioxidant activity of *Calamintha glandulosa* (Req.) Bentham. J. Sci. Food Agric..

[101] Öztürk G., Yilmaz G., Gülnur E.K.Ş.İ., Demitri B. (2021). Chemical composition and antibacterial activity of *Clinopodium nepeta* subsp. *glandulosum* (Req.) Govaerts essential oil. Nat. Vol. Essent. Oils.

[102] Alan S., Kürkçüoglu M., Hüsnü Can Baser K. (2011). Composition of Essential Oils of *Calamintha nepeta* (L.) Savi subsp. nepeta and *Calamintha nepeta* (L.) Savi subsp. *glandulosa* (Req.) P.W. Ball. Asian J. Chem..

[103] De Pooter H.L., Goetghebeur P., Schamp N. (1987). Variability in composition of the essential oil of *Calamintha nepeta*. Phytochemistry.

[104] Ristorcelli D., Tomi F., Casanova J. (1996). Essential oils of *Calamintha nepeta* subsp. *nepeta* and subsp. *glandulosa* from Corsica (France). J. Essent. Oil Res..

[105] Kizil S., Ipek A., Arslan N., Khawar K.M. (2008). Effect of different developing stages on some agronomical characteristics and essential oil composition of oregano (*Oreganum onites*). N. Zeal. J. Crop Hortic. Sci..

[106] Lukas B., Schmiderer C., Novak J. (2015). Essential oil diversity of European *Origanum vulgare* L. (Lamiaceae). Phytochemistry.

[107] Yurteri E., Seyis F., Küplemez H. Investigation of the essential oil components of *Calamintha nepeta* (L.) savi. subsp. *glandulosa* PLANT. Proceedings of the International Baku Scientific Reserch Congress.

[108] Hussain A.I., Anwar F., Nigam P.S., Ashraf M., Gilani A.H. (2010). Seasonal variation in content, chemical composition and antimicrobial and cytotoxic activities of essential oils from four *Mentha* species. J. Sci. Food Agric..

[109] Pacifico S., Galasso S., Piccolella S., Kretschmer N., Pan S.P., Marciano S., Bauer R., Monaco P. (2015). Seasonal variation in phenolic composition and antioxidant and anti-inflammatory activities of *Calamintha nepeta* (L.). Savi. Food Res. Int..

[110] Villa-Ruano N., Pacheco-Hernández Y., Cruz-Durán R., Lozoya-Gloria E. (2015). Volatiles and seasonal variation of the essential oil composition from the leaves of *Clinopodium macrostemum* var. *laevigatum* and its biological activities. Ind. Crops Prod..

[111] Yesil Celiktas O., Hames Kocabas E.E., Bedir E., Vardar Sukan F., Ozek T., Baser K.H.C. (2007). Antimicrobial activities of methanol extracts and essential oils of *Rosmarinus officinalis*, depending on location and seasonal variations. Food Chem..

[112] Atti-Santos A.C., Pansera M.R., Paroul N., Atti-Serafini L., Moyna P. (2004). Seasonal variation of essential oil yield and composition of *Thymus vulgaris* L. (Lamiaceae) from South Brazil. J. Essent. Oil Res..

[113] McGimpsey J.A., Douglas M.H., Van Klink J.W., Beauregard D.A., Perry N.B. (1994). Seasonal variation in essential oil yield and composition from naturalized *Thymus vulgaris* L. in New Zealand. Flavour Fragr. J..

[114] Chebel A.V., Koroch A.R., Juliani J.R., Juliani H.R., Trippi V.S. (1998). Micropropagation of Minthostachys mollis (H.B.K.) Grieseb. and essential oil composition of clonally propagated plants. Vitr. Cell. Dev. Biol.-Plant.

[115] Fortunato M.I., Avato P. (2008). Plant development and synthesis of essential oils in micropropagated and mycorrhiza inoculated plants of *Origanum vulgare* L. ssp. *hirtum* (Link) Ietswaart. Plant Cell Tiss. Org. Cult..

[116] Kuźma Ł., Kalemba D., Różalski M., Różalska B., Więckowska-Szakiel M., Krajewska U., Wysokińska H. (2009). Chemical composition and biological activities of essential oil from *Salvia sclarea* plants regenerated in vitro. Molecules.

[117] Skała E., Kalemba D., Wajs A., Różalski M., Krajewska U., Różalska B., Więckowska-Szakiel M., Wysokińska H. (2007). In vitro propagation and chemical and biological studies of the essential oil of *Salvia przewalskii* Maxim. Z. Für Naturforschung.

[118] Argyropoulou C., Daferera D., Tarantilis P.A., Fasseas C., Polissiou M. (2007). Chemical composition of the essential oil from leaves of *Lippia citriodora* H.B.K. at two developmental stages. Biochem. Syst. Ecol..

[119] Prins C.L., Vieira I.J.C., Freitas S.P. (2010). Growth regulators and essential oil production. Braz. J. Plant Physiol..

[120] Ioannidis D., Bonner L., Johnson C.B. (2002). UV-B is Required for Normal Development of Oil Glands in *Ocimum basilicum* L. (Sweet Basil). Ann. Bot..

[121] Schreiner M., Mewis I., Huyskens-Keil S., Jansen M.A.K., Zrenner R., Winkler J.B., O’Brien N., Krumbein A. (2012). UV-B-Induced Secondary Plant Metabolites—Potential Benefits for Plant and Human Health. Crit. Rev. Plant Sci..

[122] Pandey A., Agrawal M., Agrawal S.B. (2023). Ultraviolet-B and Heavy Metal-Induced Regulation of Secondary Metabolites in Medicinal Plants: A Review. Metabolites.

[123] Murashige T., Skoog F. (1962). A revised medium for rapid growth and bioassays with tobacco tissue cultures. Physiol. Plant..

[124] Adams R.P. (2007). Identification of Essential Oil Components by Gas Chromatography/Mass Spectrometry.

[125] Lucero M., Estell R., Tellez M., Fredricksona E.D. (2009). Retention Index Calculator Simplifies Identification of Plant Volatile Organic Compounds. Phytochem. Anal..

